# Changes in predicted lean body mass, appendicular skeletal muscle mass, and body fat mass and cardiovascular disease

**DOI:** 10.1002/jcsm.12962

**Published:** 2022-02-25

**Authors:** Seong Rae Kim, Gyeongsil Lee, Seulggie Choi, Yun Hwan Oh, Joung Sik Son, Minseon Park, Sang Min Park

**Affiliations:** ^1^ Department of Dermatology Seoul National University College of Medicine Seoul South Korea; ^2^ Department of Family Medicine Seoul National University Hospital, Seoul National University College of Medicine Seoul South Korea; ^3^ Department of Biomedical Sciences Seoul National University Graduate School Seoul South Korea; ^4^ Department of Family Medicine Jeju National University School of Medicine Jeju South Korea; ^5^ Department of Family Medicine Jeju National University Hospital Jeju South Korea

**Keywords:** Lean body mass, Appendicular skeletal muscle mass, Body fat mass, Cardiovascular disease, Young adults

## Abstract

**Background:**

Little is known about the association of changes in two body components, muscle and fat mass, with the risk of cardiovascular disease (CVD) among young adults. We investigated the association of changes in predicted lean body mass index (LBMI), appendicular skeletal muscle mass index (ASMI), and body fat mass index (BFMI) with the development of CVD among young adults.

**Methods:**

This nationwide, population‐based cohort study included 3 727 738 young adults [2 406 046 (64.5%) men and 1 321 692 (35.5%) women] aged 20–39 years without a previous history of CVD who underwent two health screening examinations during 2009–2010 and 2011–2012. Using validated and robust prediction equations, we calculated the changes in predicted LBMI, ASMI, and BFMI from the first to the second examinations.

**Results:**

The mean (SD) age was 32.2 (4.9) years, and 2 406 046 (64.5%) of the participants were men. A total of 23 344 CVD events were detected during 22 257 632 person‐years of follow‐up. Each 1 kg/m^2^ increase in predicted LBMI and ASMI change was associated with a reduced risk of CVD among men [adjusted hazard ratio (aHR): 0.86, 95% confidence interval (CI) 0.82–0.91; aHR: 0.76, 95% CI 0.69–0.82, respectively] and women (aHR: 0.77, 95% CI 0.63–0.95; aHR: 0.75, 95% CI 0.59–0.96). Each 1 kg/m^2^ increase in predicted BFMI change was associated with an increased risk of CVD among men (aHR: 1.16, 95% CI 1.10–1.22) and women (aHR: 1.32, 95% CI 1.06–1.65). In both sexes, decreases in predicted LBMI and ASMI were associated with greater CVD risk, and decreased predicted BFMI was associated with a reduced CVD risk. Those who maintained their BMI between −1 and +1 kg/m^2^ also had a decreased risk of CVD per 1 kg/m^2^ increase in predicted LBMI and ASMI change among men (aHR: 0.86, 95% CI 0.80–0.92; aHR: 0.85, 95% CI 0.76–0.95) and women (aHR: 0.62, 95% CI 0.47–0.83; aHR: 0.59, 95% CI 0.44–0.80) and had a greater risk of CVD per 1 kg/m^2^ increase in predicted BFMI change among men (aHR: 1.17, 95% CI 1.10–1.25) and women (aHR: 1.64, 95% CI 1.20–2.23). Regardless of changes in weight, such as from normal to obese or vice versa, these results were consistent.

**Conclusions:**

Among young adults, increased predicted muscle mass or decreased predicted fat mass were associated with a reduced risk of development of CVD. Decreased predicted muscle mass or increased predicted fat mass were associated with an elevated risk of development of CVD.

## Introduction

Obesity, an excessive accumulation of body fat, is a main risk factor for the development of cardiovascular disease (CVD).^1^, ^2^, ^3^ Increasing body mass index (BMI) or weight gain is detrimental to CVD.^4^, ^5^, ^6^, ^7^ However, the effects of decreasing BMI or weight loss on the risk of CVD are still controversial as protective,^6^ not significant,^5^, ^7^ and even hazardous.^4^, ^8^

One possible explanation for the unexpectedly detrimental effect of weight loss on CVD is related to the tools for assessing obesity. The surrogate markers of obesity used in large‐scale studies were anthropometric values, including BMI, waist or hip circumference, and skinfold thickness, which cannot distinguish fat mass from muscle mass, despite their different physiological effects. In order to accurately examine the association of changes in body composition with CVD, it is imperative to distinguish between fat and muscle with regard to body composition and identify the physiological effects of them on CVD.

Although there are some accurate measurements of body composition that distinguish fat and muscle mass, such as dual‐energy X‐ray absorptiometry (DXA), computed tomography scan, and magnetic resonance imaging,^9^, ^10^ these modalities have critical limitations to their use in large‐scale epidemiologic studies due to their high cost and the time required. Therefore, we developed and validated robust prediction equations for body fat mass (BFM), lean body mass (LBM), and appendicular skeletal muscle mass (ASM) using anthropometric measures, allowing us to estimate fat and muscle mass and determine the independent effects of these two different body components on CVD.^11^ Using these prediction equations, we aimed to investigate the association between changes in predicted fat and muscle mass, considered separately, and CVD development in young adults aged 20–39 years.

## Methods

### Study population

The participants in this population‐based cohort study were derived from the National Health Insurance Service (NHIS) database. The NHIS provides a wide range of mandatory health insurance coverage to 97% of the Korean people and collects data including hospital utilization, medical treatment, and pharmaceutical drug prescriptions.^12^, ^13^ Information on sociodemographic characteristics, blood tests, and health behaviours is also collected from biennial health screening examinations provided by NHIS for all employed and self‐employed insured people aged 20 years or older.^13^ A variety of epidemiologic studies have used the NHIS data for research purposes.^12^

**Figure 1:**
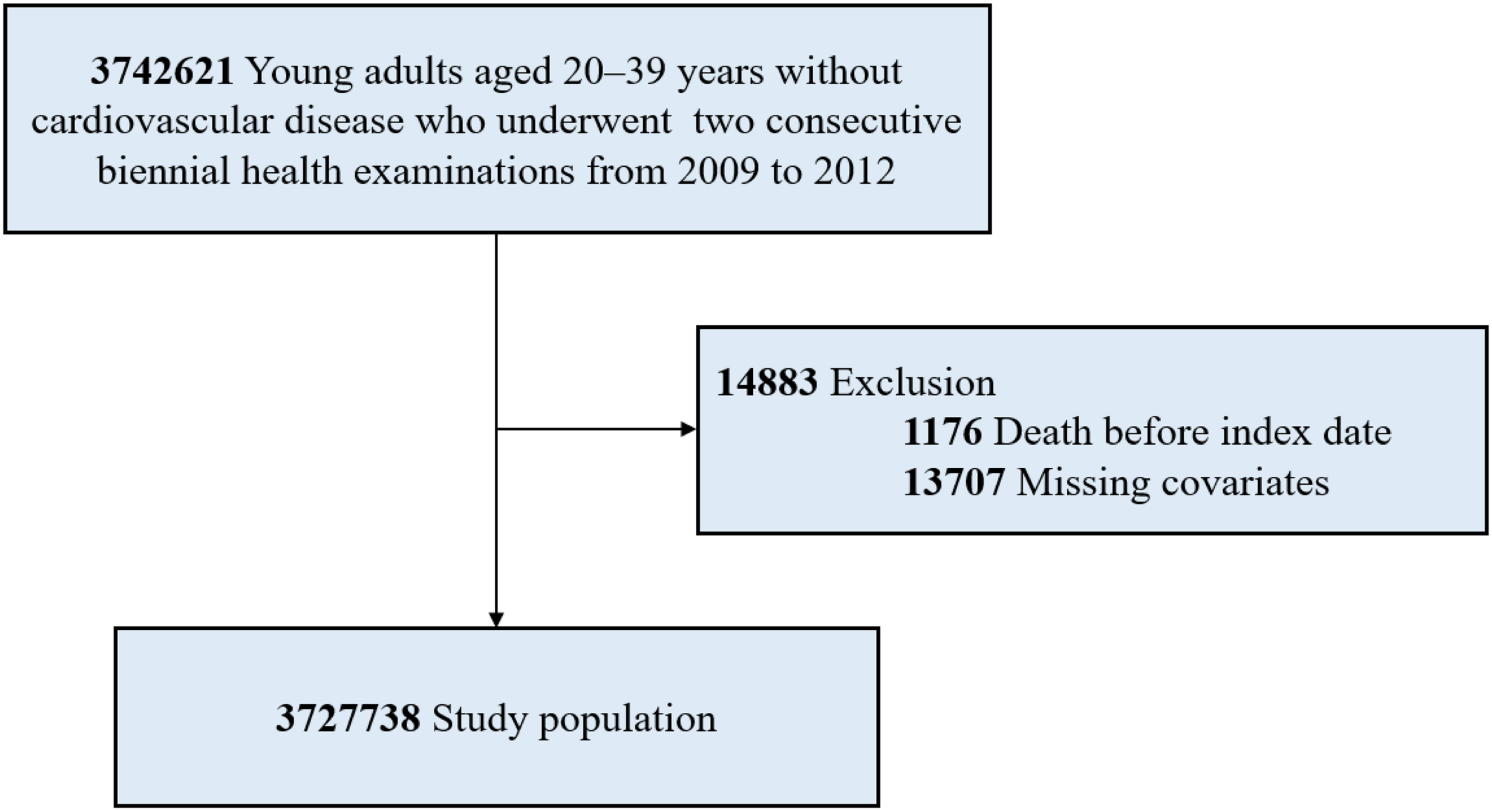
Flow diagram of selection of study population.

Among 3 742 621 young adults aged 20 to 39 years without a previous history of CVD who underwent two health screening examinations during 2009–2010 and 2011–2012, data from 1176 individuals who were dead and 13 707 individuals without data on covariates before the index date of 1 January 2013 were excluded. Finally, a total of 3 727 738 [2 406 046 (64.5%) men and 1 321 692 (35.5%) women] participants were included and followed up from 1 January 2013 to 31 December 2018 (*Figure* 1).

### Assessment of lean body mass, appendicular skeletal muscle mass, and fat mass

The development and validation of predicted LBM, ASM, and BFM, which are applicable to Koreans, are described in a previous study.^11^ In brief, using a representative sample of the Korean population including 7599 men and 10 009 women from the Korean National Health and Nutrition Examination Survey, we performed multivariable linear regression to derive the prediction equations for LBM, ASM, and BFM using the following data: LBM, ASM, and BFM from whole‐body DXA as dependent variables and age, anthropometric measurements (height, body weight, and waist circumference), laboratory tests (serum creatinine level), and health behaviours (physical activity, smoking status, and alcohol intake) as independent variables that are possible factors affecting body composition. The prediction equations were validated through the Bland–Altman plot and intraclass correlation coefficient (ICC) in the independent validation group. The final prediction equations (Supporting Information, *Table* S1) showed a fairly high predictive power for LBM (for men, adjusted *R* ^2^ = 0.86, standard error of estimate 2.67 kg; for women, adjusted *R* ^2^ = 0.79, standard error of estimate 2.18 kg), ASM (for men, adjusted *R* ^2^ = 0.81, standard error of estimate 1.56 kg; for women, adjusted *R* ^2^ = 0.72, standard error of estimate 1.20 kg), and BFM (for men, adjusted *R* ^2^ = 0.75, standard error of estimate 2.70 kg; for women, adjusted *R* ^2^ = 0.83, standard error of estimate 2.24 kg). Based on the Bland–Altman plot in the previous study,^11^ the 95% limits of agreement between each actual mass index and predicted mass index would be approximately estimated to be ±1–1.5 kg/m^2^. Furthermore, all equations showed low bias and moderate agreement from the Bland–Altman plot and high ICC indicating a good agreement between actual and predicted values when applied in a large‐scale population study.^11^

Based on these robust prediction equations, we calculated the predicted lean body mass index (LBMI), appendicular skeletal muscle mass index (ASMI), and body fat mass index (BFMI) of each participant using data on age, anthropometric measurements, laboratory tests, and health behaviours from the two consecutive biennial health screening examinations during the first (2009–2010) and second (2011–2012) periods in this cohort study. Each predicted value is computed as LBM, ASM, and BFM in kilograms divided by the height in metres squared. The changes in each predicted value were determined by subtracting each predicted value obtained during the first health examination from that obtained during the second health examination.

### Follow‐up for cardiovascular disease

We collected hospital admission records from the NHIS along with codes from the International Classification of Diseases, Tenth Revision (ICD‐10) to determine CVD events, including coronary heart disease (CHD) and stroke, which occurred between 1 January 2013 and 31 December 2018. According to the American Heart Association guidelines, we used ICD‐10 codes to identify CVD (ICD‐10 codes I20–I25, I60–I69), CHD (ICD‐10 codes I20–I25), and stroke (ICD‐10 codes I60–I69).^14^ To rule out events that were not actual CVD, we defined CVD events as 2 or more days of hospitalization with ICD‐10 codes for CVD.

### Statistical analysis

Each participant included in this cohort was followed up starting on 1 January 2013 and censored at the CVD event, death, or 31 December 2018 whichever happened first. To assess the hazard ratios and 95% confidence intervals (95% CIs) for CVD, including CHD and stroke, depending on changes in predicted LBMI, ASMI, and BFMI between two consecutive health examinations, we performed Cox proportional hazard regression analysis. We also presented restricted cubic splines of changes in predicted LBMI, ASMI, and BFMI to visually assess the association of the changes in predicted LBMI, ASMI, and BFMI with CVD, including CHD and stroke.^15^ According to studies, four knots were placed at the 5th, 35th, 65th, and 95th percentiles of the change in predicted LBMI, ASMI, and BFMI, respectively.^16^, ^17^ In addition, we conducted subgroup analyses stratifying by age, physical activity, alcohol intake, smoking status, Charlson comorbidity index (CCI), systolic blood pressure, fasting serum glucose, and total cholesterol.

Main analyses by the Cox proportional hazards regression models were adjusted for the following potential confounding factors: baseline predicted value (continuous: kg/m^2^), age (continuous: years), sex (categorical: male or female), household income (categorical: 1st, 2nd, 3rd, or 4th quartile), physical activity (categorical: 0, 1–2, 3–4, or ≥5 times per week), smoking status (categorical: never, past, or current smoker), alcohol intake (categorical: 0, 1–2, 3–4, or ≥5 times per week), baseline and secondary BMI (continuous: kg/m^2^), systolic blood pressure (continuous: mmHg), fasting serum glucose (continuous: mg/dL), total cholesterol (continuous: mg/dL), and CCI (continuous). In addition, we developed an additional multivariable model after adjusting all potential confounding factors except for variables used in the prediction equations to derive predicted values, including age, physical activity, smoking status, and alcohol intake. Household income was estimated by the insurance premium of each participant. Physical activity, smoking status, and alcohol intake were assessed by a self‐reported questionnaire from the health screening check‐up. The CCI was assessed by an algorithm according to the previous study.^18^ We defined two‐sided *P* < 0.05 as indicating statistical significance. We performed all analyses, data collection, and data mining using SAS Version 9.4 (SAS Institute, Cary, NC, USA) and R programming Version 3.3.3 (the R Foundation for Statistical Computing, Vienna, Austria).

## Results

During up to 22 257 632 person‐years of follow‐up, a total of 23 344 CVD events were identified. *Table* 1 shows the descriptive characteristics of all participants in this study. Among 3 727 738 participants, the mean (SD) age was 32.2 (4.9) years, and 2 406 046 (64.5%) were men. The mean (SD) BMI was 24.20 (3.29) for men and 21.31 (3.14) for women at the first health examination and increased in both men and women at the second health examination. Men tended to have lower predicted BFMI and higher predicted LBMI and ASMI than women in both health screening examinations.

**Table 1:** General characteristics of the study population

	Total	Men	Women
Number of people	3 727 738	2 406 046	1 321 692
Health screening examination Period I (2009–2010)			
BMI, kg/m^2^, mean (SD)	23.18 (3.52)	24.20 (3.29)	21.31 (3.14)
Predicted LBMI, kg/m^2^, mean (SD)	17.12 (2.54)	18.55 (1.80)	14.51 (1.31)
Predicted ASMI, kg/m^2^, mean (SD)	7.51 (1.36)	8.36 (0.82)	5.98 (0.60)
Predicted BFMI, kg/m^2^, mean (SD)	5.82 (1.72)	5.40 (1.50)	6.59 (1.81)
Health screening examination Period II (2011–2012)			
BMI, kg/m^2^, mean (SD)	23.44 (3.59)	24.49 (3.32)	21.55 (3.26)
Predicted LBMI, kg/m^2^, mean (SD)	17.25 (2.59)	18.72 (1.81)	14.58 (1.35)
Predicted ASMI, kg/m^2^, mean (SD)	7.55 (1.38)	8.41 (0.82)	5.98 (0.59)
Predicted BFMI, kg/m^2^, mean (SD)	5.95 (1.76)	5.50 (1.52)	6.75 (1.88)
Age, mean (SD)	32.2 (4.9)	32.9 (4.6)	31.0 (5.2)
Household income, quartile, *N* (%)			
1st (highest)	963 906 (25.8)	746 993 (31.1)	216 913 (16.4)
2nd	1 371 533 (36.8)	940 052 (39.1)	431 481 (32.7)
3rd	852 904 (22.9)	454 372 (18.8)	398 532 (30.2)
4th (lowest)	539 395 (14.5)	264 629 (11.0)	274 766 (20.7)
Physical activity, times per week, *N* (%)			
0	1 494 300 (40.1)	855 433 (35.6)	638 867 (48.3)
1–2	1 015 467 (27.2)	678 277 (28.2)	337 190 (25.5)
3–4	617 055 (16.6)	430 888 (17.9)	186 167 (14.1)
≥5	600 916 (16.1)	441 448 (18.3)	159 468 (12.1)
Smoking, *N* (%)			
Never	1 933 345 (51.9)	696 559 (29.0)	1 236 786 (95.6)
Former	512 159 (13.7)	475 101 (19.8)	37 058 (2.8)
Current	1 282 234 (34.4)	1 234 386 (51.2)	47 848 (3.6)
Alcohol intake, times per week, *N* (%)			
0	1 332 453 (35.8)	581 877 (24.2)	750 576 (56.8)
1–2	1 916 805 (51.4)	1 414 580 (58.8)	502 225 (38.0)
3–4	407 543 (10.9)	347 571 (14.5)	59 972 (4.5)
≥5	70 937 (1.9)	62 018 (2.5)	8919 (0.7)
Systolic blood pressure, mmHg, mean (SD)	118.4 (13.3)	122.4 (12.4)	111.2 (11.7)
Fasting serum glucose, mg/dL, mean (SD)	92.3 (17.2)	94.3 (18.9)	88.6 (13.0)
Total cholesterol, mg/dL, mean (SD)	189.7 (34.8)	194.4 (35.4)	181.1 (31.9)
Estimated glomerular filtration rate, mL/min/1.73 m^2^, *N* (%)			
≥90	1 478 781 (54.5)	946 991 (52.4)	531 790 (58.5)
60–90	1 213 181 (44.7)	845 704 (46.9)	367 477 (40.5)
30–60	18 662 (0.7)	10 226 (0.6)	8436 (0.9)
<30	1681 (0.1)	1161 (0.1)	520 (0.1)
Charlson comorbidity index, *N* (%)			
0	2 313 948 (62.0)	1 554 550 (64.6)	759 398 (57.5)
1	1 124 597 (30.2)	681 318 (28.3)	443 279 (33.5)
≥2	289 193 (7.8)	170 178 (7.1)	119 015 (9.0)

ASMI, appendicular skeletal muscle mass index; BFMI, body fat mass index; BMI, body mass index; LBMI, lean body mass index; *N*, the number of people; SD, standard deviation.

**Figure 2:**
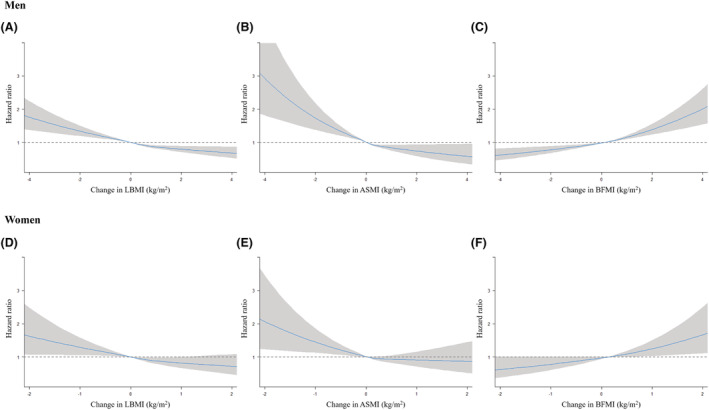
Association of the changes in predicted lean body mass index (LBMI), appendicular skeletal muscle mass index (ASMI), and body fat mass index (BFMI) with cardiovascular disease among young adults. Solid lines indicate hazard ratio and the shaded regions show the 95% confidence intervals from restricted cubic spline regression. Restricted cubic splines were constructed with four knots placed at the 5th, 35th, 65th, and 95th percentiles of the change in predicted LBMI, ASMI, and BFMI. Hazard ratios (95% confidence interval) were calculated by Cox proportional hazards regression analysis after adjusting for each baseline predicted value, age, household income, baseline and secondary body mass index, physical activity, smoking status, alcohol intake, systolic blood pressure, fasting serum glucose, total cholesterol, and Charlson comorbidity index.

*Figure* 2 shows that an increase in predicted LBMI was associated with a reduced risk of CVD in both sexes, although the risk‐reducing effect on CVD was slightly attenuated statistically in women. An increase in predicted ASMI was also associated with a decreased risk of CVD in men, whereas the risk‐reducing effect of ASMI on CVD was statistically insignificant in women. A decrease in predicted LBMI and ASMI was associated with an increased risk of CVD in both sexes. Furthermore, an increase in predicted BFMI was associated with an increased risk of CVD in both sexes, whereas a decrease in predicted BFMI was associated with a reduced risk of CVD in both sexes. The association of changes in predicted LBMI, ASMI, and BFMI with respect to CHD and stroke presented similar results and trends (*Figures* S1 and S2).

The associations of changes in predicted LBMI, ASMI, and BFMI with the risk of CVD stratified by baseline weight status are presented in *Table* 2 as Model 1 and Model 2. Overall in Model 1, each 1 kg/m^2^ increase in predicted LBMI and ASMI change was associated with a decreased risk of CVD among men [LBMI, adjusted hazard ratio (aHR): 0.86, 95% CI 0.82–0.91, *P* < 0.001; ASMI, aHR: 0.76, 95% CI 0.69–0.82, *P* < 0.001] and women (LBMI, aHR: 0.77, 95% CI 0.63–0.95, *P* < 0.01; ASMI, aHR: 0.75, 95% CI 0.59–0.96, *P* < 0.01). In contrast, each 1 kg/m^2^ increase in BFMI change was associated with an increased risk of CVD among men (aHR: 1.16, 95% CI 1.10–1.22, *P* < 0.001) and women (aHR: 1.32, 95% CI 1.06–1.65, *P* < 0.01). For men who were normal weight (BMI 18.5–22.9), overweight (BMI 23–24.9), and obese (BMI ≥ 25) at baseline, the associations of the increase in each predicted value with CVD risk showed similar results and trends, whereas the statistical significance of those associations was not detected in women by weight stratification. The results from Model 2 were also consistent with the main findings presented in Model 1, except for those of the associations of the increase in LBMI or BFMI with CVD risk in women.

**Table 2:** Hazard ratios and 95% confidence intervals of cardiovascular disease per 1 kg/m^2^ increase in change in predicted lean body mass index, appendicular skeletal muscle mass index, and body fat mass index stratified by the baseline weight status among young adults

Baseline weight status	Events, *N*	Person‐years	aHR (95% CI) for CVD per 1 kg/m^2^ increase in each predicted value					
			Model 1^a^			Model 2^b^		
			LBMI	ASMI	BFMI	LBMI	ASMI	BFMI
Male participants								
Overall	18 692	14 351 637	0.86 (0.82–0.91)***	0.76 (0.69–0.82)***	1.16 (1.10–1.22)***	0.92 (0.88–0.97)***	0.60 (0.55–0.65)***	1.06 (1.02–1.11)**
Normal (BMI 18.5–22.9)	4394	5 024 462	0.84 (0.75–0.94)**	0.66 (0.55–0.81)***	1.20 (1.07–1.34)**	0.90 (0.81–0.99)*	0.47 (0.39–0.56)***	1.10 (0.99–1.21)
Overweight (BMI 23–24.9)	4333	3 636 764	0.84 (0.75–0.94)**	0.73 (0.60–0.89)**	1.19 (1.06–1.33)**	0.92 (0.83–1.02)	0.59 (0.49–0.70)***	1.07 (0.97–1.18)
Obese (BMI ≥ 25)	9724	5 366 768	0.87 (0.82–0.93)***	0.78 (0.70–0.88)***	1.15 (1.07–1.23)***	0.93 (0.87–0.98)*	0.64 (0.58–0.71)***	1.06 (1.00–1.12)*
Female participants								
Overall	4652	7 905 995	0.77 (0.63–0.95)**	0.75 (0.59–0.96)**	1.32 (1.06–1.65)**	1.13 (0.97–1.30)	0.43 (0.34–0.53)***	0.87 (0.74–1.04)
Normal (BMI 18.5–22.9)	2559	4 946 787	0.85 (0.64–1.13)	0.76 (0.55–1.06)	1.19 (0.88–1.62)	1.18 (0.96–1.45)	0.35 (0.26–0.48)***	0.82 (0.65–1.04)
Overweight (BMI 23–24.9)	702	926 814	0.59 (0.34–1.02)	0.57 (0.29–1.12)	1.60 (0.90–2.82)	1.07 (0.73–1.57)	0.56 (0.32–0.99)*	0.91 (0.59–1.40)
Obese (BMI ≥ 25)	948	885 807	0.70 (0.46–1.06)	0.78 (0.47–1.29)	1.45 (0.94–2.25)	0.97 (0.71–1.34)	0.50 (0.32–0.78)**	1.02 (0.72–1.45)

aHR, adjusted hazard ratio; ASMI, appendicular skeletal muscle mass index; BFMI, body fat mass index; BMI, body mass index; CI, confidence interval; CVD, cardiovascular disease; LBMI, lean body mass index; *N*, the number of people.

^a^ aHR (95% CI) were calculated by Cox hazards regression analysis after adjusting for baseline each predictor, age, household income, baseline and secondary BMI, physical activity, smoking status, alcohol intake, systolic blood pressure, fasting serum glucose, total cholesterol, and Charlson comorbidity index.

^b^ aHR (95% CI) were calculated by Cox hazards regression analysis after adjusting for baseline each predictor, household income, baseline and secondary BMI, systolic blood pressure, fasting serum glucose, total cholesterol, and Charlson comorbidity index.

* *P* < 0.05.

** *P* < 0.01.

*** *P* < 0.001.

*Table* 3 shows the association of changes in predicted LBMI, ASMI, and BFMI with risk of CVD according to the change in weight status in Model 1 and Model 2. Participants who maintained their weight stably (change in BMI ranging between −1 and +1 kg/m^2^) had a reduced risk of CVD per 1 kg/m^2^ increase in predicted LBMI and ASMI change for men (LBMI, aHR: 0.86, 95% CI 0.80–0.92, *P* < 0.001; ASMI, aHR: 0.85, 95% CI 0.76–0.95, *P* < 0.001) and women (LBMI, aHR: 0.62, 95% CI 0.47–0.83, *P* < 0.001; ASMI, aHR: 0.59, 95% CI 0.44–0.80, *P* < 0.001), whereas they had an elevated risk of CVD per 1 kg/m^2^ increase in predicted BFMI change among men (aHR: 1.17, 95% CI 1.10–1.25) and women (aHR: 1.64, 95% CI 1.20–2.23, *P* < 0.001) in Model 1. In particular, even for men whose weight increased from normal to obese or overweight, a 1 kg/m^2^ increase in predicted LBMI and ASMI change still appeared to be associated with a reduced risk of CVD, whereas a 1 kg/m^2^ increase in predicted BFMI change appeared to be associated with an elevated risk of CVD, despite the statistical insignificance. In addition, even for men whose weight decreased from obese or overweight to normal, a 1 kg/m^2^ increase in predicted BFMI change still seemed to be associated with an increased risk of CVD, whereas a 1 kg/m^2^ increase in predicted LBMI and ASMI change seemed to be associated with a decreased risk of CVD, despite the statistical insignificance. Generally, these results and trends were maintained in men, regardless of changes in weight status, whereas those were statistically insignificant in women by changes in weight status. The trends from Model 2 were similar with the main results in Model 1 in men and women.

**Table 3:** Hazard ratios and 95% confidence intervals of cardiovascular disease per 1 kg/m^2^ increase in change in predicted lean body mass index, appendicular skeletal muscle mass index, and body fat mass index by change in weight status among young adults

Category	Events, *N*	Person‐years	aHR (95% CI) for CVD per 1 kg/m^2^ increase in each predicted value					
			Model 1^a^			Model 2^b^		
			LBMI	ASMI	BFMI	LBMI	ASMI	BFMI
Male participants								
Weight stable (change in BMI ± 1)^c^	11 634	8 903 004	0.86 (0.80–0.92)***	0.85 (0.76–0.95)***	1.17 (1.10–1.25)***	0.92 (0.87–0.98)**	0.58 (0.52–0.64)***	1.07 (1.00–1.13)*
Normal (BMI 18.5–22.9) at health examination Period I (2009–2010)								
Continuously normal	3335	3 860 125	0.93 (0.87–0.99)*	0.77 (0.61–0.96)*	1.10 (0.97–1.26)	0.95 (0.84–1.07)	0.51 (0.42–0.63)***	1.03 (0.92–1.16)
Normal to overweight (BMI 23–24.9)	910	984 226	0.65 (0.52–0.82)***	0.41 (0.27–0.63)***	1.56 (1.24–1.96)***	0.72 (0.59–0.89)**	0.33 (0.23–0.49)***	1.36 (1.10–1.67)**
Normal to obese (BMI ≥ 25)	85	100 592	0.64 (0.35–1.18)	0.39 (0.13–1.17)	1.60 (0.87–2.93)	0.79 (0.45–1.39)	0.36 (0.13–0.99)*	1.26 (0.72–2.20)
Overweight (BMI 23–24.9) at health examination Period I (2009–2010)								
Overweight to normal (BMI 18.5–22.9)	589	523 321	0.79 (0.59–1.07)	0.62 (0.37–1.03)	1.27 (0.94–1.72)	0.86 (0.66–1.14)	0.47 (0.30–0.74)**	1.13 (0.86–1.48)
Continuously overweight	2515	2 136 041	0.88 (0.75–1.02)	0.73 (0.56–0.94)*	1.15 (0.98–1.34)	0.96 (0.84–1.11)	0.60 (0.47–0.76)***	1.02 (0.89–1.17)
Overweight to obese (BMI ≥ 25)	1228	976 610	0.82 (0.67–1.00)*	0.83 (0.58–1.17)	1.21 (0.99–1.49)	0.90 (0.75–1.09)	0.67 (0.49–0.92)*	1.08 (0.90–1.30)
Obese (BMI ≥ 25) at health examination Period I (2009–2010)								
Obese to normal (BMI 18.5–22.9)	56	44 671	0.57 (0.30–1.11)	0.47 (0.16–1.42)	1.74 (0.89–3.40)	0.49 (0.28–0.87)*	0.37 (0.14–0.97)*	1.99 (1.13–3.49)*
Obese to overweight (BMI 23–24.9)	836	551 260	0.94 (0.73–1.21)	0.96 (0.63–1.47)	1.07 (0.83–1.38)	0.92 (0.74–1.15)	0.61 (0.42–0.89)*	1.07 (0.86–1.33)
Continuously obese	8830	4 770 059	0.87 (0.81–0.93)***	0.77 (0.67–0.87)***	1.15 (1.08–1.24)***	0.93 (0.87–0.99)*	0.64 (0.58–0.72)***	1.06 (0.99–1.12)
Female participants								
Weight stable (change in BMI ± 1)^c^	2740	4 861 533	0.62 (0.47–0.83)***	0.59 (0.44–0.80)***	1.64 (1.20–2.23)***	0.98 (0.78–1.22)	0.33 (0.24–0.44)***	1.03 (0.81–1.32)
Normal (BMI 18.5–22.9) at health examination Period I (2009–2010)								
Continuously normal	2118	4 163 000	0.85 (0.61–1.16)	0.79 (0.55–1.13)	1.23 (0.86–1.74)	1.14 (0.90–1.46)	0.34 (0.24–0.48)***	0.86 (0.65–1.13)
Normal to overweight (BMI 23–24.9)	264	426 646	0.89 (0.40–1.97)	0.55 (0.19–1.53)	1.18 (0.50–2.79)	1.32 (0.90–1.94)	0.33 (0.13–0.80)*	0.72 (0.43–1.20)
Normal to obese (BMI ≥ 25)	51	80 919	1.14 (0.26–4.99)	1.08 (0.16–7.44)	0.84 (0.18–4.00)	1.06 (0.29–3.91)	0.87 (0.14–5.24)	0.88 (0.22–3.50)
Overweight (BMI 23–24.9) at health examination Period I (2009–2010)								
Overweight to normal (BMI 18.5–22.9)	171	262 693	0.72 (0.22–2.31)	0.55 (0.13–2.28)	1.41 (0.41–4.92)	1.65 (0.85–3.20)	0.65 (0.19–2.20)	0.56 (0.23–1.34)
Continuously overweight	326	437 508	0.45 (0.20–1.02)	0.42 (0.15–1.16)	2.09 (0.88–4.98)	0.85 (0.45–1.59)	0.43 (0.19–0.98)*	1.17 (0.60–2.31)
Overweight to obese (BMI ≥ 25)	205	224 903	0.82 (0.34–1.99)	0.97 (0.39–2.43)	1.17 (0.46–2.94)	1.11 (0.59–2.07)	0.98 (0.41–2.33)	0.84 (0.42–1.68)
Obese (BMI ≥ 25) at health examination Period I (2009–2010)								
Obese to normal (BMI 18.5–22.9)	28	46 149	ND	ND	ND	ND	ND	ND
Obese to overweight (BMI 23–24.9)	100	129 074	0.45 (0.10–1.94)	0.42 (0.07–2.63)	2.85 (0.61–13.3)	1.02 (0.36–2.89)	0.24 (0.06–1.02)	1.09 (0.33–3.56)
Continuously obese	820	709 941	0.68 (0.43–1.05)	0.78 (0.46–1.33)	1.50 (0.94–2.39)	0.92 (0.65–1.31)	0.52 (0.32–0.84)**	1.09 (0.75–1.59)

aHR, adjusted hazard ratio; ASMI, appendicular skeletal muscle mass index; BFMI, body fat mass index; BMI, body mass index; CI, confidence interval; CVD, cardiovascular disease; LBMI, lean body mass index; *N*, the number of people; ND, not determined.

^a^ aHR (95% CI) were calculated by Cox hazards regression analysis after adjusting for baseline each predictor, age, household income, baseline and secondary BMI, physical activity, smoking status, alcohol intake, systolic blood pressure, fasting serum glucose, total cholesterol, and Charlson comorbidity index.

^b^ aHR (95% CI) were calculated by Cox hazards regression analysis after adjusting for baseline each predictor, household income, baseline and secondary BMI, systolic blood pressure, fasting serum glucose, total cholesterol, and Charlson comorbidity index.

^c^ Participants with change in BMI ranging between −1 and +1 kg/m^2^ in the second health examination (2009–2010) compared with the first health examination (2011–2012).

* *P* < 0.05.

** *P* < 0.01.

*** *P* < 0.001.

The results of subgroup analyses stratified by age, physical activity, alcohol intake, smoking status, comorbidity (CCI), systolic blood pressure, fasting serum glucose, and total cholesterol are given in *Table* S2. For men, the results and trends of association of changes in predicted LBMI, ASMI, and BFMI with CVD risk in most subgroups were consistent with the overall results. For women, the results of association of changes in predicted LBMI, ASMI, and BFMI with CVD risk were statistically significant in relatively older or healthier female group such as those who are physically active, non‐drinker, never smoker, with CCI 0, with systolic blood pressure < 130 mmHg, with fasting serum glucose < 126 mg/dL, or with total cholesterol < 200 mg/dL.

## Discussion

In this large‐scale cohort study, we found that increased predicted LBMI and ASMI, representing muscle mass, or decreased predicted BFMI, representing fat mass, were associated with a reduced risk of the development of CVD. Conversely, decreased predicted LBMI and ASMI or increased predicted BFMI were associated with an elevated risk of the development of CVD. These trends were maintained regardless of changes in weight, such as from normal weight to obese or from obese to normal weight. To our knowledge, this is the first study to identify the association of changes in body composition, by separating muscle mass and fat mass as predicted LBMI, ASMI, and BFMI, with the development of CVD, instead of using BMI or body weight as a composite of muscle and fat mass.

Although there have been no previous studies investigating the association of changes in muscle or fat mass with CVD, multiple studies have shown an association between changes in BMI or body weight, as a composite of fat and muscle mass, and the risk of CVD. Generally, increasing BMI and weight gain have been consistently reported to elevate the risk of CVD. One study showed that each 5 kg/m^2^ increase in BMI was related to a 30% increase in the risk of CVD mortality among adults aged 30–59 years.^4^ The results from the Nurses' Health Study and US men in the Health Professionals Follow‐Up Study cohorts showed that weight gain was significantly associated with increased risk of incident CVD.^5^ In addition, weight gain in early adulthood was associated with increased risk of CVD in midlife.^6^, ^7^ In the present study, in particular, we found that increasing fat mass was related to an elevated risk of CVD among young adults.

The physiologic impact of obesity on the development of CVD has been well reviewed in previous studies. Excessive fat accumulation activates the renin‐angiotensin‐aldosterone system and the sympathetic nervous system, followed by hypertension, which is the major risk factor for CVD.^19^ In addition, fat tissue acts as an endocrine organ that synthesizes diverse proinflammatory cytokines, which cause diastolic dysfunction, and adipokines, which cause insulin resistance, endothelial dysfunction, and subsequent atherosclerosis.^20^, ^21^ From a macroscopic viewpoint, obesity leads to sleep apnoea and hypoventilation syndrome, followed by hypoxia and acidosis, which causes subsequent pulmonary atrial hypertension.^1^ Taken together, excessive fat or increasing body weight may be a risk factor for CVD by causing neurohormonal activation and cardiovascular dysregulation, which partly supports our findings.

However, the effects of decreasing BMI or weight loss on CVD risk are still unclear. Although multiple studies reported that reduced BMI was associated with a decreased risk of CVD in young adults,^6^ some previous studies found that the association between weight loss and CVD development or mortality was not significant,^5^, ^7^ and some studies even reported that weight loss was associated with an increased risk of CVD or mortality,^4^, ^8^, ^22^ which is frequently observed in older adults and patients with chronic diseases. One possible reason that weight loss failed to have a protective effect on the primary prevention of CVD may be explained based on our results that decreasing fat mass and decreasing muscle mass have different effects on the development of CVD. Unlike weight gain, which is usually due to an increase in fat mass, weight loss may have two causes: purposeful weight loss, which is mainly due to decrease in fat mass, and unintended weight loss, which is mainly due to decreased muscle mass. In our current study, we distinguished fat and muscle mass and found that decreased fat mass was significantly associated with a reduced risk of CVD development, and decreased muscle mass was significantly associated with an elevated risk of CVD development. These results imply that one of the major reasons for the unexpectedly negative effect of weight loss on CVD might be due to the loss of muscle mass more than the loss of fat mass, which means that simply losing weight is not always beneficial to prevent CVD.

Increasing muscle mass with purposeful weight loss has many benefits to cardiovascular health. Increasing muscle mass through regular exercise with purposeful weight loss contributes to reducing total blood volume, stroke volume, preload, left ventricular filling pressure, and subsequent cardiac output.^20^ At the microscopic level, myokines released from skeletal muscle have anti‐inflammatory effects.^23^ These mechanisms are plausible, in that increased muscle mass was associated with a reduced risk of CVD development in the current study. In particular, we showed that the benefits of increased muscle mass on cardiovascular health were maintained even in individuals with increased BMI or weight gain, which suggests the importance of muscle mass instead of simple BMI values to prevent incident CVD. However, the benefits of increase in muscle mass to cardiovascular health were statistically less noticeable in women than in men. This may be attributable to the relatively small sample size of women in this cohort, which is half the sample size of the men.

The associations of changes in predicted LBMI, ASMI, and BFMI with CVD risk were statistically significant in the older or healthier female groups. The relatively larger number of CVD events of older women than younger women may contribute the statistical significance because those in their 30s are at high risk of CVD compared with those in their 20s. Furthermore, it can be assumed that CVD prevention benefits can be remarkably obtained when healthy women increase muscle mass and decrease fat mass. Meanwhile, the association of the change in predicted ASMI, especially an increase in ASMI, with stroke was not statistically significant in women. Our results are somewhat in line with the previous study reporting that the risk‐reducing effect of increased muscle mass against stroke event was obvious in men, but not women.^24^ The authors failed to explain accurately the underlying mechanism of the phenomenon, but we guess that excessive physical activity or heavy resistance training to increase in muscle mass may affect stroke event in women. According to the previous studies, excessive physical activity or heavy lifting, which may contribute to platelet aggregation and transient elevation in blood pressure, were associated with greater risk of stroke.^25^, ^26^ Considering that it is generally more difficult for women to gain muscle mass than men, women have to engage in physical activity and resistance training far more than men to increase the same amount of absolute muscle mass, represented as ASMI, which could rather weaken the cerebrovascular health benefits of ASMI. Further studies are necessary to accurately identify the association of the change in predicted ASMI, especially an increase in ASMI, with stroke in young women. Ultimately, appropriate level of purposeful interventions to increase muscle mass and decrease fat mass, including healthy diet strategies or regular aerobic and resistance trainings, may be beneficial for young adults to prevent the CVD development.

It would not be feasible to use our prediction equations, which are applicable to Koreans, directly in different ethnic populations; however, our study outcomes suggest that by comparing with other pre‐established prediction equations, our prediction equations can be applied to other races. First, most pre‐established prediction equations were developed using anthropometric measurements that were easily accessible in each country in common.^27^, ^28^, ^29^, ^30^, ^31^ In this study, other possible factors affecting body composition, such as laboratory test results (serum creatinine level) and health behaviours (physical activity, smoking status, and alcohol intake), were additionally used to enhance the predictive power of prediction equations. Second, unlike some studies, we validated our prediction equations in terms of both absolute (using ICC) and relative (using the Bland–Altman plot) reliability to guarantee the trustworthiness of results from the equations. Lastly, we also derived the prediction equations by separating men and women to enhance the reliability of prediction equations. One study developed prediction equations without separating men and women, which resulted in proportional errors of the absolute reliability, including underprediction of the body composition of men and overprediction of that of women.^29^ A recent study from the USA investigating the association between predicted LBM and BFM and mortality in men somewhat complemented the aforementioned limitations and showed results similar to those of our study,^16^ although it did not consider laboratory test results or health behaviours. It should be possible to develop a more reliable and comprehensive prediction equations for various ethnic populations by considering the aforementioned important points and deriving prediction equations using large sample including multiple races.

This study has some strengths. First, we calculated LBM, ASM, and BFM separately using robust and validated anthropometric prediction equations. Although these attempts have been made before,^27^, ^29^, ^30^, ^31^, ^32^ there has been no study to estimate ASM, which is regarded as an especially useful indicator for assessing the effects of total skeletal muscle, because the muscles of the extremities account for more than 75% of total skeletal muscle and reflect the change in muscle mass well.^33^, ^34^ Moreover, this is the first nationwide large cohort study to examine the independent effects of changes in LBM, ASM, and BFM on CVD among young adults. Second, the study is based on a large population of about 3.74 million young men and women, which increases the reliability and generalizability of the results. Finally, the study was adjusted for a variety of health behaviours and metabolic mediators of CVD, such as physical activity, smoking status, alcohol consumption, blood pressure, serum glucose, total cholesterol, and comorbidities, which enhances the reliability of our findings.

This study has several limitations. First, the predicted LBMI, ASMI, and BFMI were not precise values of actual LBMI, ASMI, and BFMI. Nevertheless, the prediction equations showed a high predictive power, and the validation results of the prediction equations showed low bias and high ICC to assess LBMI, ASMI, and BFMI in the same ethnic group.^11^ Second, due to the nature of retrospective cohort study design, we did not fully consider confounding factors. However, this nationwide database was comprehensive, and most subjects were of the same ethnic group and from a relatively narrow age band of 20 to 39 years. Finally, the predicted equations were not validated for the changes in the body components, although they were well validated for single measures. However, the classification errors caused by prediction equations may be reduced as we estimated the changes in each variable of same person. In addition, according to the previous study using the same prediction equations, an increase in the relative LBM and ASM was associated with the lower risk of metabolic syndrome, whereas an increase in the relative BFM was associated with the greater risk of metabolic syndrome,^35^ which is in line with our findings. Considering the metabolic syndrome is a well‐known major risk factor for CVD, our findings may be sufficiently reliable. However, further validation is required to elevate reliability by collecting longitudinal LBM, ASM, and BFM data from whole‐body DXA for the same individual.

## Conclusions

Among young adults, an increase in muscle mass, represented as predicted LBMI and ASMI, or a decrease in fat mass, represented as predicted BFMI, was associated with a reduction in the risk of developing CVD. Conversely, a decrease in muscle mass or an increase in fat mass was associated with a significant elevation in the risk of developing CVD. Understanding the independent effects of LBM, ASM, and BFM, instead of simply focusing on BMI, provides novel insights into the effects of body weight change on CVD. Increasing muscle mass and decreasing fat mass through purposeful intervention, including nutrition or exercise intervention strategies, may help young adults to decrease the risk of developing CVD.

## Conflict of interest

None declared.

## Funding

This research was supported by a grant of the MD–PhD/Medical Scientist Training Program through the Korea Health Industry Development Institute (KHIDI), funded by the Ministry of Health and Welfare, Republic of Korea.

## Ethical approval

This study was approved by the Institutional Review Board (IRB) at the Seoul National University Hospital (IRB number: 1703‐039‐836) and has therefore been performed in accordance with the ethical standards laid down in the 1964 Declaration of Helsinki and its later amendments. All participants were informed of the objective of the survey, and they provided their consent. The NHIS database was anonymized according to strict confidentiality guidelines prior to distribution by the NHIS.

## Supporting information


**Table S1.** Anthropometric prediction equations for lean body mass, appendicular skeletal muscle mass, and body fat mass
**Table S2.** Subgroup analyses of the association of changes in predicted lean body mass index, appendicular skeletal muscle mass index, and body fat mass index with subsequent cardiovascular disease among young adults.
**Figure S1.** Association of the changes in predicted lean body mass index, appendicular skeletal muscle mass index, and body fat mass index with coronary heart disease among young adults. Solid lines indicate hazard ratio and the shaded regions show the 95% confidence intervals from restricted cubic spline regression. Restricted cubic splines were constructed with four knots placed at the 5th, 35th, 65th, and 95th percentiles of the change in predicted LBMI, ASMI, and BFMI. HRs (95% CI) were calculated by Cox proportional hazards regression analysis after adjusting for each baseline predicted value, age, household income, baseline and secondary BMI, physical activity, smoking status, alcohol intake, systolic blood pressure, fasting serum glucose, total cholesterol, and Charlson comorbidity index. *BMI* Body mass index; *LBMI* Lean body mass index; *ASMI* Appendicular skeletal muscle mass index; *BFMI* Body fat mass index; *HR* hazard ratio; *CI* Confidence interval.
**Figure S2.** Association of the changes in predicted lean body mass index, appendicular skeletal muscle mass index, and body fat mass index with stroke among young adults. Solid lines indicate hazard ratio and the shaded regions show the 95% confidence intervals from restricted cubic spline regression. Restricted cubic splines were constructed with four knots placed at the 5th, 35th, 65th, and 95th percentiles of the change in predicted LBMI, ASMI, and BFMI. HRs (95% CI) were calculated by Cox proportional hazards regression analysis after adjusting for each baseline predicted value, age, household income, baseline and secondary BMI, physical activity, smoking status, alcohol intake, systolic blood pressure, fasting serum glucose, total cholesterol, and Charlson comorbidity index. *BMI* Body mass index *LBMI* Lean body mass index; *ASMI* Appendicular skeletal muscle mass index; *BFMI* Body fat mass index; *HR* hazard ratio; *CI* Confidence interval.
