# Supplementary material for: Changes in predicted lean body mass, appendicular skeletal muscle mass, and body fat mass and cardiovascular disease
Source: J Cachexia Sarcopenia Muscle. 2022 Feb 25;13(2):1113–23. doi: 10.1002/jcsm.12962 (PMC8978024; doi:10.1002/jcsm.12962)
Supplement: Supplementary file 1 — Table S1. Anthropometric prediction equations for lean body mass, appendicular skeletal muscle mass, and body fat mass Table S2. Subgroup analyses of the association of changes in predicted lean body mass index, appendicular skeletal muscle mass index, and body fat mass index with subsequent cardiovascular disease among young adults. Figure S1. Association of the changes in predicted lean body mass index, appendicular skeletal muscle mass index, and body fat mass index with coronary heart disease among young adults. Solid lines indicate hazard ratio and the shaded regions show the 95% confidence intervals from restricted cubic spline regression. Restricted cubic splines were constructed with four knots placed at the 5th, 35th, 65th, and 95th percentiles of the change in predicted LBMI, ASMI, and BFMI. HRs (95% CI) were calculated by Cox proportional hazards regression analysis after adjusting for each baseline predicted value, age, household income, baseline and secondary BMI, physical activity, smoking status, alcohol intake, systolic blood pressure, fasting serum glucose, total cholesterol, and Charlson comorbidity index. BMI Body mass index; LBMI Lean body mass index; ASMI Appendicular skeletal muscle mass index; BFMI Body fat mass index; HR hazard ratio; CI Confidence interval. Figure S2. Association of the changes in predicted lean body mass index, appendicular skeletal muscle mass index, and body fat mass index with stroke among young adults. Solid lines indicate hazard ratio and the shaded regions show the 95% confidence intervals from restricted cubic spline regression. Restricted cubic splines were constructed with four knots placed at the 5th, 35th, 65th, and 95th percentiles of the change in predicted LBMI, ASMI, and BFMI. HRs (95% CI) were calculated by Cox proportional hazards regression analysis after adjusting for each baseline predicted value, age, household income, baseline and secondary BMI, physical activity, smoking status, alcohol intake, systol [file JCSM-13-1113-s001.docx]

**SUPPLEMENTAL MATERIAL**

**Journal name**: Journal of Cachexia, Sarcopenia and Muscle

**Title**: Changes in predicted lean body mass, appendicular skeletal muscle mass, and body fat mass and cardiovascular disease

**Authors**: Seong Rae Kim*, Gyeongsil Lee*, Seulggie Choi, Yun Hwan Oh, Joung Sik Son, Minseon Park, Sang Min Park.

*Seong Rae Kim and Gyeongsil Lee contributed equally to this work.

**Corresponding author**: Sang Min Park, MD, PhD

Department of Family Medicine, Seoul National University Hospital, Seoul National University College of Medicine, 28 Yunkeon-dong, Jongro-gu, Seoul 110-744, South Korea

Tel: +82-2-2072-3331 Fax: +82-2-766-3276

E-mail address: smpark.snuh@gmail.com

**Table S1.** Anthropometric prediction equations for lean body mass, appendicular skeletal muscle mass, and body fat mass.

| Prediction equations for men^a^ | | |
| --- | --- | --- |
| LBM (kg) | -0.296 + 0.012*age (years) + 0.134*height (cm) + 0.675*weight (kg) - 0.201*waist (cm) - 0.249*serum creatinine (mg/dL) + 0.270*moderate physical activity + 0.924*vigorous physical activity – 0.559*past smoker + 0.234*current smoker - 0.046*moderate drinker + 0.324*heavy drinker | R^2^=0.86  SEE=2.67 |
| ASM (kg) | -2.236 - 0.011*age (years) + 0.081*height (cm) + 0.324*weight (kg) - 0.121*waist (cm) – 0.008*serum creatinine (mg/dL) + 0.200*moderate physical activity + 0.587*vigorous physical activity – 0.195*past smoker – 0.016*current smoker + 0.004*moderate drinker + 0.151*heavy drinker | R^2^=0.81  SEE=1.56 |
| BFM (kg) | 0.561 - 0.012*age (years) - 0.133*height (cm) + 0.310*weight (kg) + 0.199*waist (cm) + 0.253*serum creatinine (mg/dL) - 0.247*moderate physical activity - 0.878*vigorous physical activity + 0.708*past smoker - 0.275*current smoker + 0.077*moderate drinker - 0.291*heavy drinker | R^2^=0.75  SEE=2.70 |
| Prediction equations for women^a^ | | |
| LBM (kg) | -11.941 + 0.015*age (years) + 0.171*height (cm) + 0.457*weight (kg) - 0.060*waist (cm) + 0.428*serum creatinine (mg/dL) + 0.181*moderate physical activity + 0.654*vigorous physical activity – 0.254*past smoker + 0.221*current smoker + 0.209*moderate drinker + 0.126*heavy drinker | R^2^=0.79  SEE=2.18 |
| ASM (kg) | -8.447 + 0.002*age (years) + 0.091*height (cm) + 0.203*weight (kg) - 0.034*waist (cm) + 0.539*serum creatinine (mg/dL) + 0.103*moderate physical activity + 0.362*vigorous physical activity – 0.203*past smoker + 0.000*current smoker + 0.103*moderate drinker + 0.031*heavy drinker | R^2^=0.72  SEE=1.20 |
| BFM (kg) | 12.269 - 0.014*age (years) - 0.172*height (cm) + 0.530*weight (kg) + 0.058*waist (cm) – 0.314*serum creatinine (mg/dL) - 0.123*moderate physical activity - 0.541*vigorous physical activity + 0.261*past smoker - 0.242*current smoker - 0.218*moderate drinker - 0.182*heavy drinker | R^2^=0.83  SEE=2.24 |

^a^ The prediction equations were derived and validated using the Korean National Health and Nutrition Examination Survey 2008–2011.

Physical activity, smoking, and alcohol intake are binary variables (yes=1; no=0) as follows:

Moderate physical activity: those who engage in 600-2999 METs-min/week of physical activity;

Vigorous physical activity: those who engage in ≥3000 METs-min/week of physical activity;

Moderate drinker: drinking alcohol less than 14 drinks per week (men), or less than 7 drinks per week (women);

Heavy drinker: drinking alcohol 14 drinks or more per week (men), or 7 drinks or more per week (women).

*LBM* Lean body mass; *ASM* Appendicular skeletal muscle mass; *BFM* Body fat mass; *SEE* standard error of estimate; *MET* Metabolic Equivalent Task.

**Table S2.** Subgroup analyses of the association of changes in predicted lean body mass index, appendicular skeletal muscle mass index, and body fat mass index with subsequent cardiovascular disease among young adults.

|  | aHR (95% CI) for CVD per 1 kg/m^2^ increase in change in each predicted value | | |
| --- | --- | --- | --- |
| Subgroup | LBMI | ASMI | BFMI |
| **Male participants** |  |  |  |
| **Age** |  |  |  |
| 20-29 | 0.87 (0.75-1.01) | **0.77 (0.60-0.99)^*^** | 1.14 (0.98-1.33) |
| 30-39 | **0.86 (0.82-0.91)^***^** | **0.76 (0.69-0.83)^***^** | **1.16 (1.10-1.22)^***^** |
| **Physical Activity** |  |  |  |
| Yes | **0.84 (0.79-0.90)^***^** | **0.71 (0.64-0.80)^***^** | **1.19 (1.11-1.27)^***^** |
| No | **0.90 (0.83-0.98)^**^** | **0.83 (0.72-0.95)^**^** | **1.12 (1.03-1.22)^*^** |
| **Alcohol Intake** |  |  |  |
| Yes | **0.85 (0.80-0.90)^***^** | **0.74 (0.67-0.82)^***^** | **1.18 (1.11-1.25)^***^** |
| No | 0.91 (0.82-1.01) | **0.82 (0.69-0.98)^*^** | 1.10 (0.99-1.22) |
| **Cigarette Smoking** |  |  |  |
| Never | **0.88 (0.79-0.99)^*^** | **0.80 (0.66-0.97)^*^** | **1.13 (1.01-1.27)^*^** |
| Past-smoker | **0.80 (0.72-0.90)^**^** | **0.66 (0.54-0.81)^**^** | **1.26 (1.12-1.41)^**^** |
| Current smoker | **0.88 (0.82-0.94)^*^** | **0.77 (0.69-0.86)^**^** | **1.14 (1.07-1.22)^*^** |
| **Charlson Comorbidity Index** |  |  |  |
| 0 | **0.88 (0.82-0.94)^**^** | **0.77 (0.68-0.86)^***^** | **1.14 (1.07-1.22)^**^** |
| ≥1 | **0.85 (0.79-0.92)^***^** | **0.75 (0.66-0.86)^***^** | **1.19 (1.09-1.27)^**^** |
| **Systolic Blood Pressure** |  |  |  |
| <130 mmHg | **0.87 (0.81-0.93)^**^** | **0.77 (0.68-0.87)^***^** | **1.15 (1.07-1.24)^**^** |
| ≥130 mmHg | **0.86 (0.80-0.93)^**^** | **0.75 (0.66-0.84)^***^** | **1.17 (1.09-1.25)^***^** |
| **Fasting serum glucose** |  |  |  |
| <126 mg/dL | **0.86 (0.82-0.91)^***^** | **0.75 (0.69-0.83)^***^** | **1.16 (1.10-1.22)^***^** |
| ≥126 mg/dL | 0.89 (0.74-1.07) | 0.81 (0.59-1.10) | 1.14 (0.94-1.37) |
| **Total cholesterol** |  |  |  |
| <200 mg/dL | **0.83 (0.77-0.89)^***^** | **0.69 (0.61-0.79)^***^** | **1.21 (1.12-1.30)^***^** |
| ≥200 mg/dL | **0.91 (0.84-0.97)^**^** | **0.82 (0.73-0.93)^**^** | **1.11 (1.04-1.19)^*^** |
| **Female participants** |  |  |  |
| **Age** |  |  |  |
| 20-29 | 0.92 (0.62-1.36) | 0.88 (0.56-1.38) | 1.23 (0.79-1.89) |
| 30-39 | **0.72 (0.57-0.92)^***^** | **0.72 (0.54-0.96)^*^** | **1.35 (1.05-1.75)^**^** |
| **Physical Activity** |  |  |  |
| Yes | **0.68 (0.51-0.91)^***^** | **0.67 (0.48-0.95)^**^** | **1.54 (1.12-2.11)^***^** |
| No | 0.90 (0.67-1.19) | 0.86 (0.61-1.19) | 1.11 (0.82-1.50) |
| **Alcohol Intake** |  |  |  |
| Yes | **0.45 (0.32-0.64)^***^** | **0.41 (0.27-0.63)^***^** | **2.12 (1.46-3.06)^***^** |
| No | 1.02 (0.79-1.31) | 1.03 (0.79-1.34) | 1.04 (0.78-1.39) |
| **Cigarette Smoking** |  |  |  |
| Never | **0.76 (0.62-0.95)^**^** | **0.75 (0.59-0.97)^*^** | **1.35 (1.08-1.71)^**^** |
| Past-smoker | 0.69 (0.21-2.23) | 0.57 (0.14-2.42) | 1.14 (0.34-3.77) |
| Current smoker | 0.79 (0.31-1.99) | 0.81 (0.27-2.41) | 1.19 (0.45-3.14) |
| **Charlson Comorbidity Index** |  |  |  |
| 0 | **0.72 (0.53-0.98)^*^** | **0.63 (0.43-0.91)^***^** | 1.37 (0.99-1.90) |
| ≥1 | 0.72 (0.62-1.08) | 0.87 (0.64-1.19) | 1.27 (0.94-1.72) |
| **Systolic Blood Pressure** |  |  |  |
| <130 mmHg | **0.77 (0.61-0.97)^*^** | **0.76 (0.58-0.99)^*^** | **1.32 (1.03-1.70)^*^** |
| ≥130 mmHg | 0.78 (0.50-1.22) | 0.81 (0.48-1.37) | 1.29 (0.80-2.09) |
| **Fasting serum glucose** |  |  |  |
| <126 mg/dL | **0.75 (0.61-0.93)^**^** | **0.72 (0.56-0.92)^***^** | **1.34 (1.07-1.67)^**^** |
| ≥126 mg/dL | 1.67 (0.61-4.63) | 2.35 (0.98-5.65) | 0.69 (0.21-2.19) |
| **Total cholesterol** |  |  |  |
| <200 mg/dL | **0.78 (0.61-1.00)^*^** | 0.80 (0.60-1.06) | **1.33 (1.01-1.75)^*^** |
| ≥200 mg/dL | 0.75 (0.53-1.07) | 0.68 (0.44-1.05) | 1.30 (0.90-1.88) |

aHR (95 % CI) were calculated by Cox hazards regression analysis after adjusting for baseline each predictor, age, household income, baseline and secondary BMI, physical activity, smoking status, alcohol intake, systolic blood pressure, total cholesterol, and Charlson comorbidity index.

*CVD* Cardiovascular disease; *LBMI* Lean body mass index; *ASMI* Appendicular skeletal muscle mass index; *BFMI* Body fat mass index; *aHR* Adjusted hazard ratio; *CI* Confidence interval.

^*^p<0.05

^**^p<0.01

^***^p<0.001

**Men**


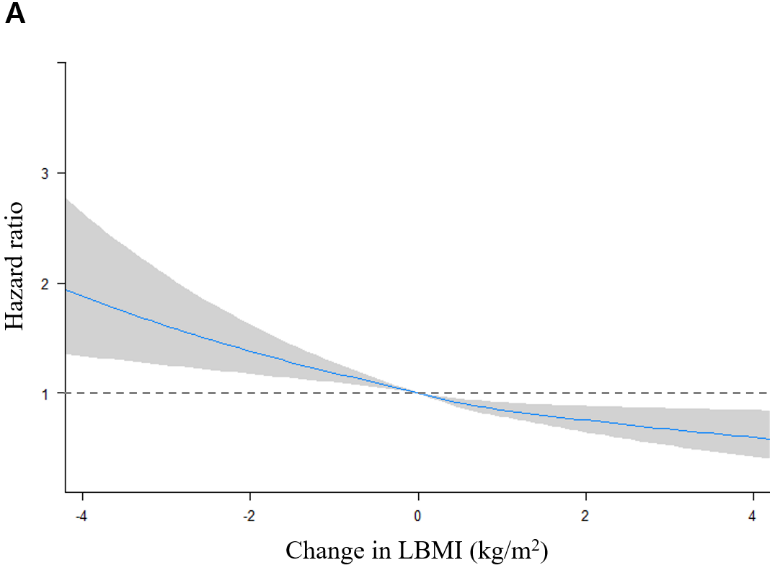

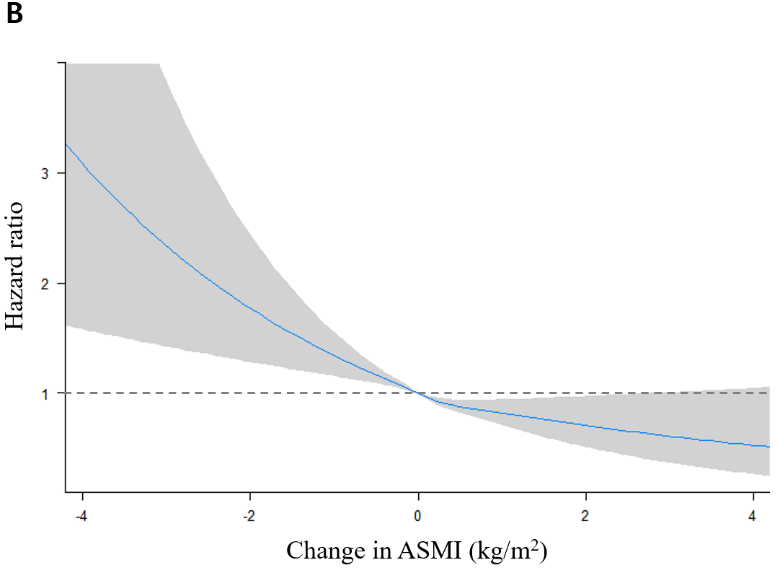

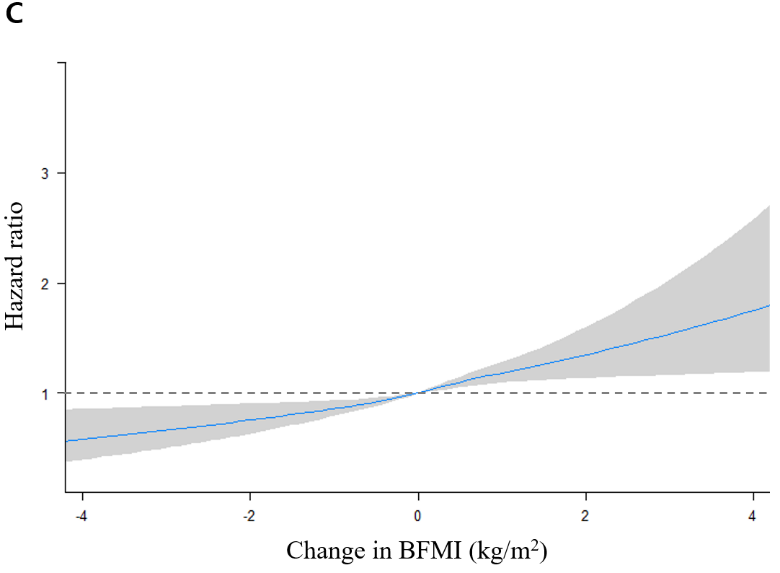


**Women**


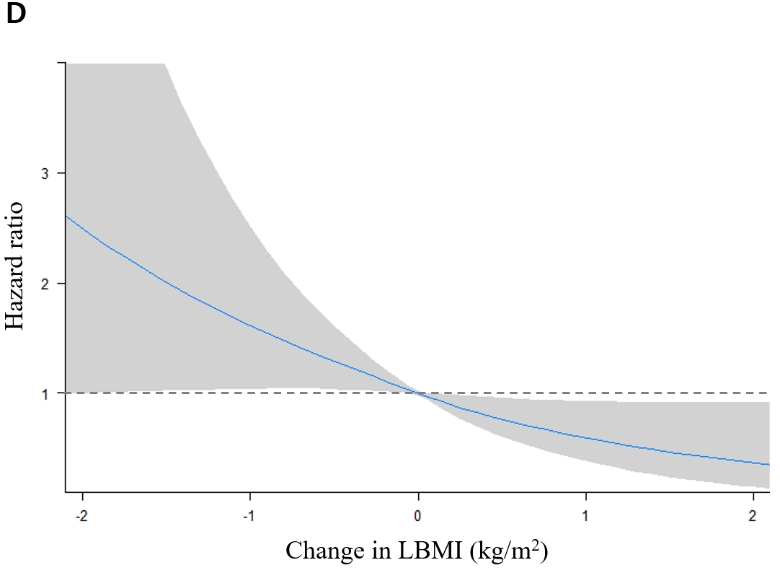

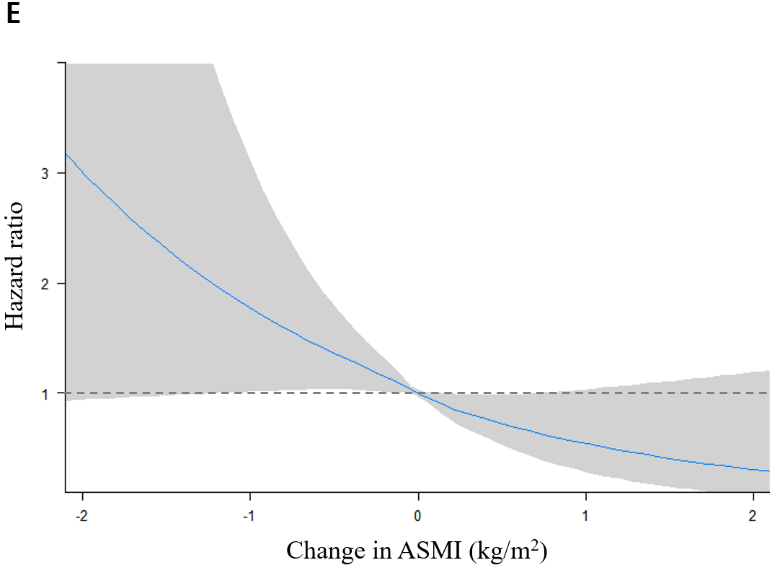

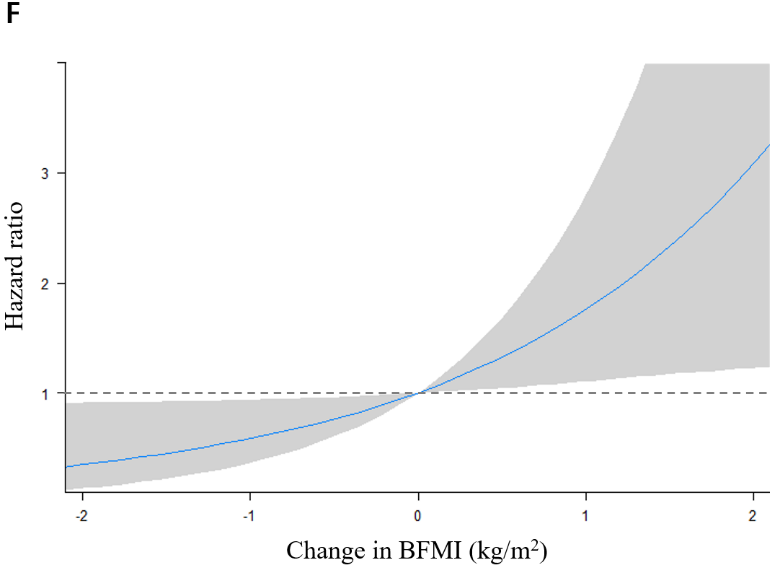
**Figure S1.** Association of the changes in predicted lean body mass index, appendicular skeletal muscle mass index, and body fat mass index with coronary heart disease among young adults. Solid lines indicate hazard ratio and the shaded regions show the 95% confidence intervals from restricted cubic spline regression. Restricted cubic splines were constructed with four knots placed at the 5th, 35th, 65th, and 95th percentiles of the change in predicted LBMI, ASMI, and BFMI. HRs (95 % CI) were calculated by Cox proportional hazards regression analysis after adjusting for each baseline predicted value, age, household income, baseline and secondary BMI, physical activity, smoking status, alcohol intake, systolic blood pressure, fasting serum glucose, total cholesterol, and Charlson comorbidity index. *BMI* Body mass index; *LBMI* Lean body mass index; *ASMI* Appendicular skeletal muscle mass index; *BFMI* Body fat mass index; *HR* hazard ratio; *CI* Confidence interval.

**Men**


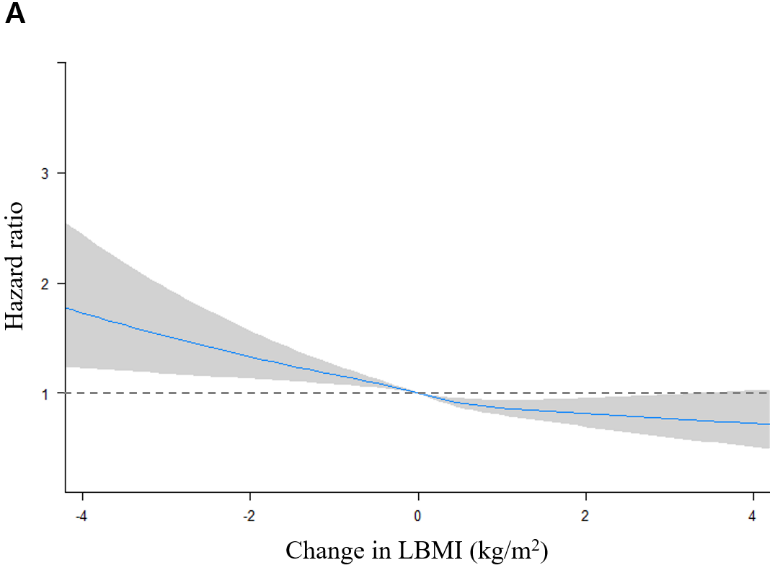

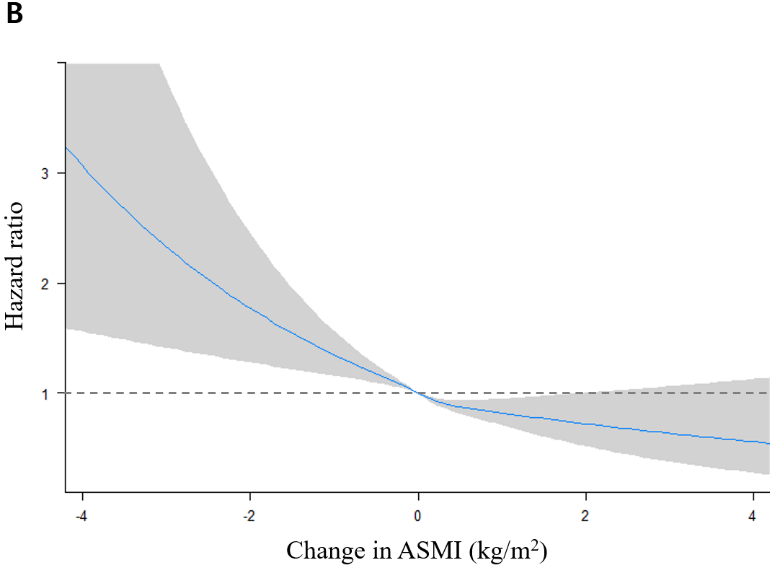

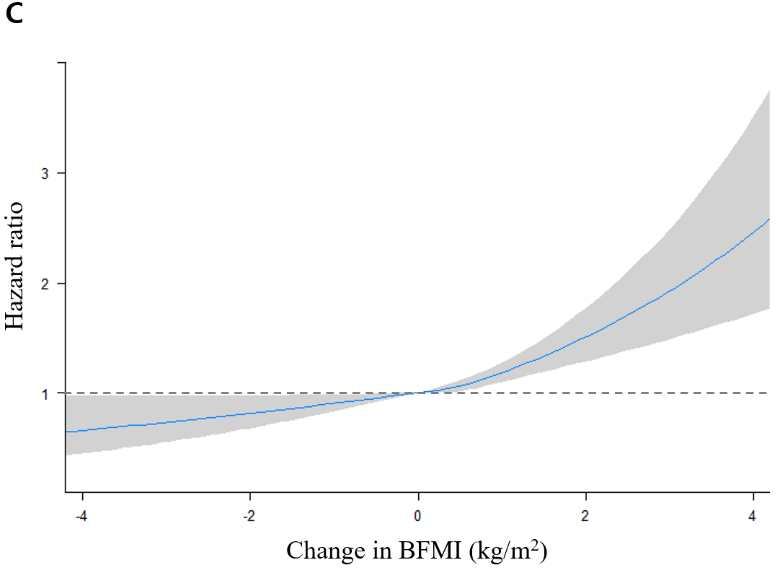


**Women**


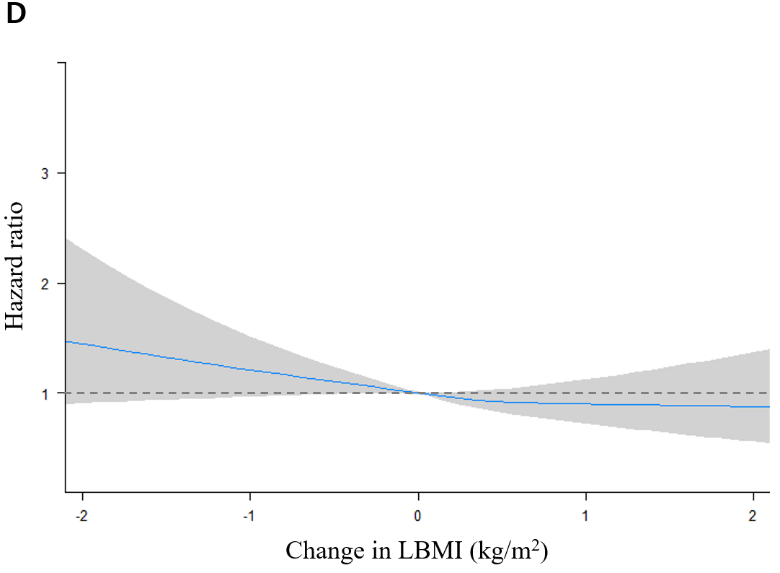

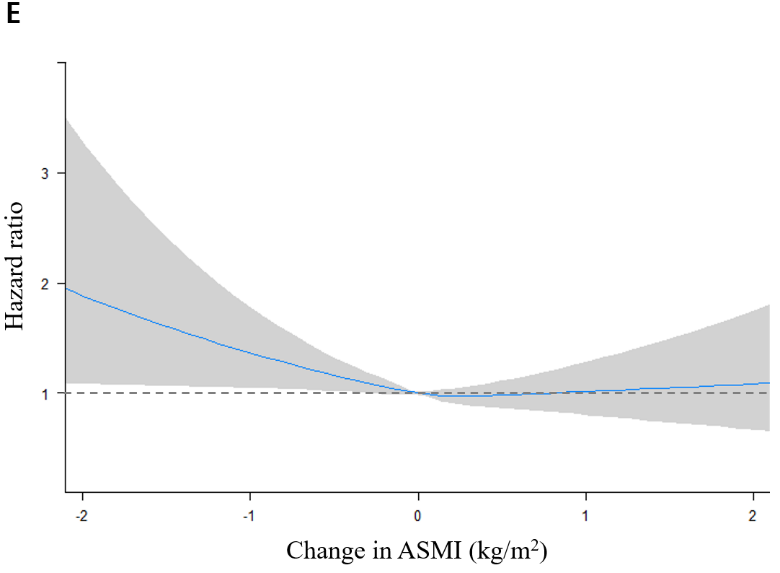

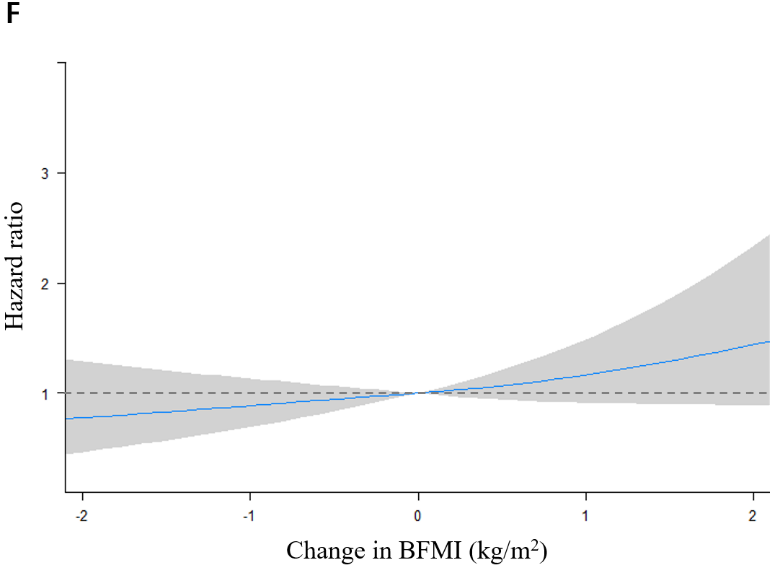
**Figure S2.** Association of the changes in predicted lean body mass index, appendicular skeletal muscle mass index, and body fat mass index with stroke among young adults. Solid lines indicate hazard ratio and the shaded regions show the 95% confidence intervals from restricted cubic spline regression. Restricted cubic splines were constructed with four knots placed at the 5th, 35th, 65th, and 95th percentiles of the change in predicted LBMI, ASMI, and BFMI. HRs (95 % CI) were calculated by Cox proportional hazards regression analysis after adjusting for each baseline predicted value, age, household income, baseline and secondary BMI, physical activity, smoking status, alcohol intake, systolic blood pressure, fasting serum glucose, total cholesterol, and Charlson comorbidity index. *BMI* Body mass index *LBMI* Lean body mass index; *ASMI* Appendicular skeletal muscle mass index; *BFMI* Body fat mass index; *HR* hazard ratio; *CI* Confidence interval.
